# A Path to Soluble Molecularly Imprinted Polymers

**DOI:** 10.3390/jfb3010001

**Published:** 2011-12-23

**Authors:** Abhilasha Verma, George M. Murray

**Affiliations:** Department of Mechanical, Aerospace and Biomedical Engineering, Center for Laser Applications, University of Tennessee Space Institute, 411 B.H. Goethert Parkway, Tullahoma, TN 37388, USA.; E-Mail: averma@utsi.edu

**Keywords:** molecular imprinting, molecularly imprinted polymers (MIPs), crosslinkable mers, organophosphates, lanthanide

## Abstract

Molecular imprinting is a technique for making a selective binding site for a specific chemical. The technique involves building a polymeric scaffold of molecular complements containing the target molecule. Subsequent removal of the target leaves a cavity with a structural “memory” of the target. Molecularly imprinted polymers (MIPs) can be employed as selective adsorbents of specific molecules or molecular functional groups. In addition, sensors for specific molecules can be made using optical transduction through lumiphores residing in the imprinted site. We have found that the use of metal ions as chromophores can improve selectivity due to selective complex formation. The combination of molecular imprinting and spectroscopic selectivity can result in sensors that are highly sensitive and nearly immune to interferences. A weakness of conventional MIPs with regard to processing is the insolubility of crosslinked polymers. Traditional MIPs are prepared either as monoliths and ground into powders or are prepared *in situ* on a support. This limits the applicability of MIPs by imposing tedious or difficult processes for their inclusion in devices. The size of the particles hinders diffusion and slows response. These weaknesses could be avoided if a means were found to prepare individual macromolecules with crosslinked binding sites with soluble linear polymeric arms. This process has been made possible by controlled free radical polymerization techniques that can form pseudo-living polymers. Modern techniques of controlled free radical polymerization allow the preparation of block copolymers with potentially crosslinkable substituents in specific locations. The inclusion of crosslinkable mers proximate to the binding complex in the core of a star polymer allows the formation of molecularly imprinted macromolecules that are soluble and processable. Due to the much shorter distance for diffusion, the polymers exhibit rapid responses. This paper reviews the methods that have been employed for the trace determination of organophosphates in real world samples using MIPs.

## 1. Introduction

Organophosphate is a term that is commonly applied to pesticides but also includes chemical warfare agents. This group of insecticides has replaced the organochlorine insecticides, such as dichlorodiphenyltrichloroethane (DDT), since they do not persist as long in the environment. Organophosphates are used in agriculture, as well as in households and gardens and by veterinarians. Such diverse applications run the risk of exposure from multiple sources that can result in toxicity. Organophosphates interfere with nerve function by impeding the enzyme acetylcholinesterase as opposed to the organochlorines that open sodium ion channels. Due to these health hazards, many countries impose strict restrictions on the organophosphate residual limits in drinking water and food. The European Union, for instance, has set the maximum allowable limit of 0.1 µg/L for individual pesticides in drinking water and 0.05 mg/kg for foods of plant origin [1]. The ability to detect such trace amounts of organophosphates is difficult and is an area of increasing concern. 

Several methods such as gas chromatography (GC), high-performance liquid chromatography (HPLC), liquid chromatography mass spectrometry (LCMS) have been employed for the detection of organophosphates in food samples [2,3,4]. However, analysis of trace amounts of organophosphates typically requires sample pretreatment, which is time consuming and can influence the accuracy and precision of the results. Thus, for the detection of organophosphates, a simple and sensitive methodology is of particular significance.

Molecular imprinting is a technique that is used for making a selective binding site for a specific molecule [5,6,7,8]. This technique involves the synthesis of a complex of the target molecule and complimentary polymerizable coordinators. Chemical and/or mechanical treatment of this complex liberates the target molecule and creates a cavity with the “memory” of the target. Molecularly imprinted polymers (MIPs) are synthetic polymers that are stable and synthesized with specific recognition sites. Due to their high selectivity, MIPs have been employed for the detection of a wide range of molecules, such as amino acids [9], pesticides [10], carbohydrates [11] and nucleic acids [12]. 

The focus of this review is on methods that have been developed for the trace determination for organophosphates in real world samples, using MIPs. The use of MIPs in organophosphate detection in pesticides is well documented [2,3,4,13,14,15,16]. However, less is known about the use of lanthanide ions in MIPs for organophosphate detection [17,18,19]. In this review, the studies developed for detection of organophosphates by discerning their effect on the luminescence of europium (III) will be discussed.

### 1.1. Chromophore

Lanthanide ions are useful as intrinsic and extrinsic chromophores. Complexation by certain organic ligands enhances the luminescence intensity of the tripositive lanthanide, Ln(III) ions. The enhancement of luminescence has been explained by a ligand to metal energy transfer mechanism. The mechanism was derived from a series of investigations by Crosby, Kasha, and their co-workers [20]. Generally, when an excited triplet state of the coordinating ligand overlaps a lanthanide electronic level, the lanthanide luminescence can be effectively pumped by a larger cross section molecular absorbance, rather than by its own weak absorbance. This process is more efficient than direct absorption of light by the lanthanide due to the poor absorptivities of the lanthanides (formally atomically forbidden absorbance for the intra-configurational f→f transition). A large number of organic ligands have been used to enhance lanthanide luminescence intensity [21]. When making an imprinted polymer sensor, the ligands must be chosen with sufficient affinity for the lanthanide, so as to coordinatively bind the ion in the polymer as well as provide intense luminescence. Many mixed ligand lanthanide complexes have been studied, providing clues to making a suitable sensor. Lanthanide ions have a thermodynamic affinity for a variety of anions, and this affinity can be exploited in making sensors for anions. Due to the relative hardness of lanthanides, the geometry of ligating atoms is a function of the steric strains imposed by the coordinating ligands. This is another avenue of exploitation for selectivity, since a careful selection of coordinating ligand can help define the line splitting by imposing specific site symmetry on the lanthanide in the resulting compound. By imposing certain coordination geometry on a complex, a large degree of change can be made to occur by ligand exchange, ensuring a significant change in spectrum upon substitution.

Luminescent lanthanide complexes provide a basis for sensing a variety of compounds. This is facilitated by the use of sensitizing ligands that provide broad-band absorbance and efficient energy transfer. β-Diketones are well known as good sensitizers for lanthanide luminescence [22]. Model systems employing β-diketone lanthanide complexes show luminescence enhancement with the addition of a variety of organophosphates. β-Diketones with fluorinated substituents were particularly effective [17,18,19]. The inclusion of β-diketones with aromatic rings was useful to shift absorbance from the UV to more easily accessible excitation wavelengths. However, in order to apply molecular imprinting as a selectivity enhancement, the ligand must be polymerizable. It was discovered that when β-diketones were functionalized with vinyl substituents, their ability to complex a lanthanide ion was often compromised. The ligand either failed to complex the lanthanide or was readily displaced whenever syntheses of an imprinting complex with an organophosphate adduct was attempted. Of the compounds prepared, only 4-vinylbenzoylmethane and 3-vinylbenzoylmethane readily formed tris complexes with europium [19]. Other vinyl-substituted β-diketones simply would not form stable complexes with europium or would be easily decomposed by addition of water. While imprinting could still be approached by first synthesizing a linear copolymer of ligand and matrix monomer, making polymer metal complexes and then mechanically cross-linking the result, such a process might make difficult the use of the polymers in certain applications.

Because of the problems found with the vinyl-substituted β-diketones, a thioester-substituted β-diketone ligand was chosen for polymerization. This approach was attractive due to the ease that vinyl-substituted β-diketones could be converted to dithioesters in a simple, one-step reaction. Further, dithioesters are useful as reagents for Reversible Addition Fragmentation Chain Transfer (RAFT) methods and are iniferters in many other organic polymerizations [23,24,25,26]. It was anticipated the formation of a dithioester ester from the vinyl substituted β-diketones would alleviate some of the unusual chemistry of the vinyl substituted β-diketones, while mitigating the Trommsdorff Effect for bulk polymerization, which has been shown to have negative implications regarding molecular imprinting [27].

### 1.2. Conventional MIPs

A weakness of conventional MIPs with regard to processing is the insolubility of crosslinked polymers. Traditional MIPs are prepared as monoliths and ground into powders or are prepared *in situ* on a support. This limits the applicability of MIPs by imposing tedious or difficult processes for their inclusion in devices. This weakness could be avoided if a means were found to prepare individual macromolecules with crosslinked binding sites but otherwise soluble linear polymers arms. Zimmerman *et al.* have prepared a type of soluble crosslinked polymer by using a porphyrin encapsulated into a crosslinked core with multiple arms to enhance solubility [28,29,30]. This process is possible due to controlled free radical polymerization techniques that can form pseudo-living polymers. Modern techniques of controlled free radical polymerization also allow the preparation of block copolymers with potentially crosslinkable substituents in specific locations. The inclusion of crosslinkable mers proximate to the binding complex in the core of a star polymer allows the formation of molecularly imprinted macromolecules that are soluble and processable.

### 1.3. Polymer Monoliths

**Figure 1:**
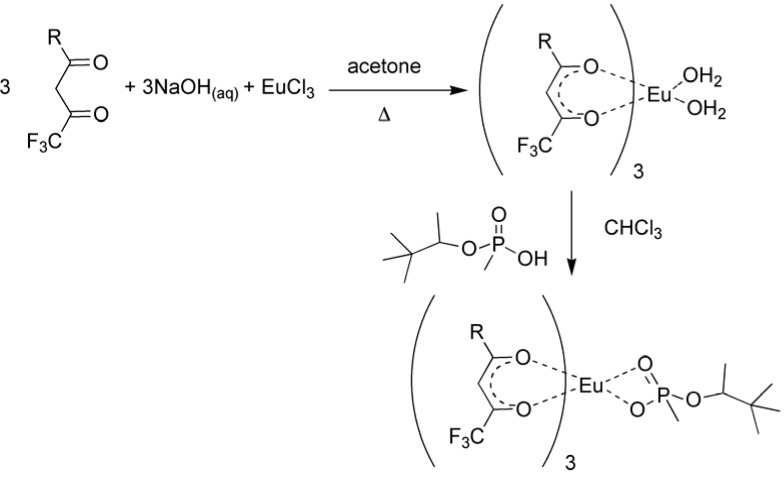
Synthesis of the tris β-diketone europium complex [17,18,19].

In order to prepare polymer monoliths it is necessary to first prepare model complexes of europium (III) with the sensitizing ligands and analyte to verify association and spectral utility. The ligands chosen were thenoyltrifluoroacetone (TTFA) and naphthoyltrifluoroacetone (NTFA) because of their high luminescent efficiencies as tris chelates with europium (III) [31,32,33,34,35,36,37]. Model complexes were prepared as diagrammed in Figure 1 [17,18,19]. The presence of water of hydration was verified using thermo-gravimetric analysis. TTFA_3_Eu had two water molecules of hydration as expected, to give europium (III) the normal coordination number of nine. NTFA_3_Eu showed an additional two molecules of water per complex that are suspected to be lattice water.

**Figure 2:**
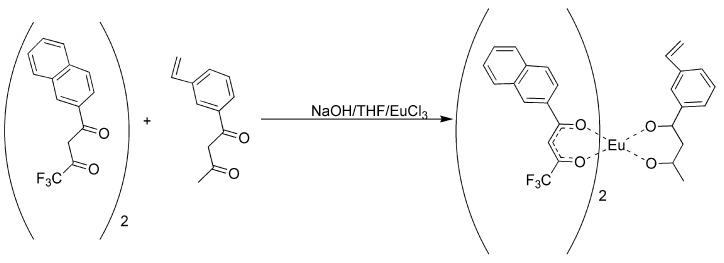
Preparation of the mixed ligand complex [17].

It was observed that the substitution of a vinyl group on the aromatic rings of the β-diketone ligands could have a negative effect upon the ligand electronics and reduce their ability to form stable complexes with europium [17,18,19]. Hence, changes in methodologies were investigated. The first and simplest method was to prepare a mixed ligand complex with two fluorinated ligands and the one vinyl-substituted β-diketone that could still complex europium (III), 3-vinyldibenzoylmethane [17]. In this manner, the fluorinated ligands made europium (III) hard enough to form phosphonate adducts and the 3-vinyldibenzoylmethane made the complex polymerizable. It was discovered that 3-vinyldibenzoylmethane could be readily displaced by pinacolylmethylphosphonate (PMP) under a number of reaction conditions. Flourinated β-diketones were found to be better ligands because of their lower polarizability, but could still be displaced by greater than equimolar amounts of PMP to complex as a free complex. The mixed ligand complexes were formed from a 2:1 ratio of naphthoyltrifluroacetone with 3-vinyldibenzoylmethane in THF with sodium hydroxide as base (Figure 2). Europium chloride was added in a minimum amount of water and the reaction was allowed to proceed for an hour. Analysis of the reaction products by elemental analysis revealed that the ligand ratio was closer to 1:1 than the original 2:1. It was thought, but not verified, that a statistical mixture of complexes had been formed. Polymers derived from the mixed ligand complexes were found to lose significant amounts of europium when washed with acetone, indicating the incomplete incorporation of the non-vinyl substituted complexes and perhaps significant amounts of the mono-vinyl substituted complexes [17]. While this mixed ligand approach was moderately successful, a process for fully incorporating complexes with at least two polymeric links was investigated.

**Figure 3:**
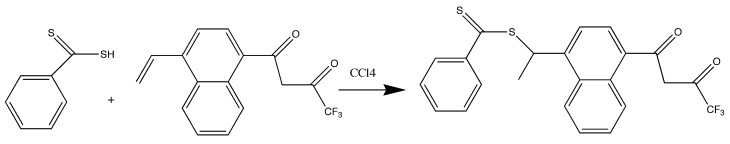
Preparation of the dithioester ligand [17,18,19].

Because of the difficulties in getting good complexation and adduct formation with vinyl substituted β-diketone ligands, an alternative approach Reversible Addition Fragmentation chain Transfer (RAFT), was employed. RAFT polymerization is a free radical process that is controlled and ensures the formation of high polymer [17,18,19,33,34,35,36,37]. The process involves a chain transfer moiety based on a dithioester. Since this functionality does not involve conjugation to the β-diketone aromatic ring, it does not interfere with the ligands’ electronics (Figure 3). Ligands prepared with the dithioester substituents seen in Figure 3 were shown to form stable complexes with europium (III) and subsequently with PMP [17,18,19].

**Figure 4:**
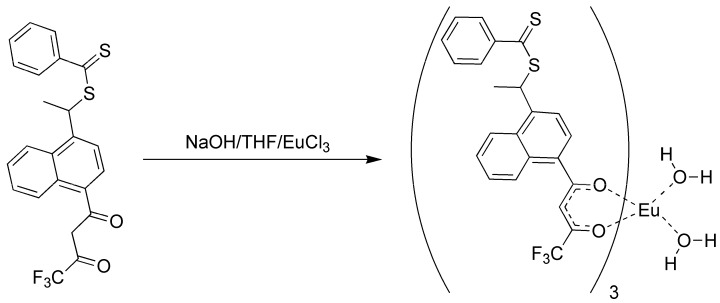
Preparation of the tris chelate dihydrate [17,18,19].

**Figure 5:**
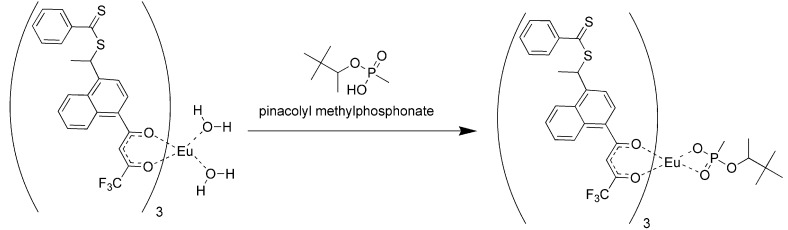
Conversion of the chelate to the imprinted complex [11].

The formation of the europium complexes and the route of adduct formation is shown in Figure 4 and Figure 5.

**Figure 6:**
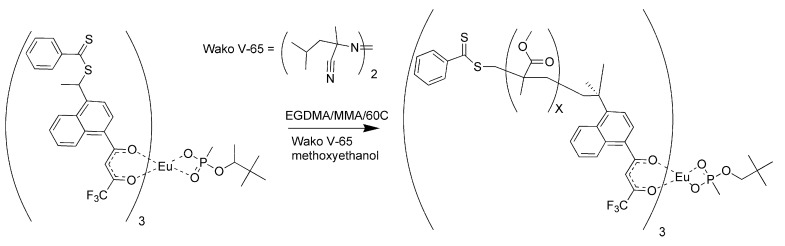
Reversible addition fragmentation chain transfer (RAFT) polymerization of the molecularly imprinted polymer (MIP) for pinacolylmethylphosphonate (PMP).

Polymers were prepared using both styrene and methyl methacrylate as matrix monomers [17]. The porosity of the polymers using toluene as solvent was limited. While toluene assured miscibility, it did not allow for phase separation as the polymer grew. The polymerizations were repeated using methoxyethanol in place of toluene as the solvent. The polymers prepared with methoxyethanol were observed to have a more open, grainy structure than those polymerized with toluene, since this highly polar solvent did allow phase separation during polymerization [17]. The polymerization procedure for the methacrylate RAFT polymer is illustrated in Figure 6.

### 1.4. Luminescence

**Figure 7:**
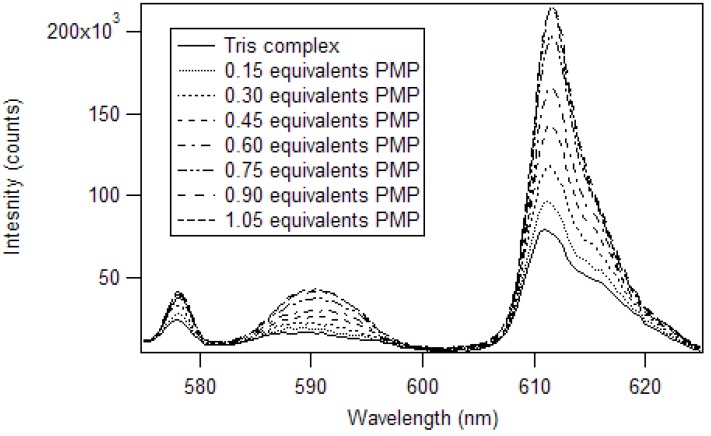
Luminescence spectra of Eu(NTFA)_3_ with addition of PMP in chloroform [17].

**Figure 8:**
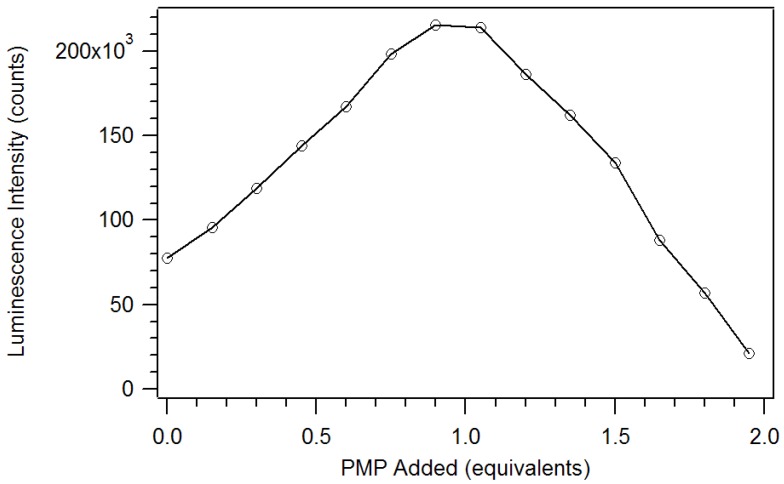
Plot of the luminescence intensity versus equivalents of PMP added [17].

Harry Brittain [38] developed a luminescence titration method for the determination of the stoichiometry of europium (III) β-diketone complexes and phosphate esters. In his work, adducts were formed in a 1:1 mole ratio for tris europium (III) β-diketone complexes, having at least one trifluoromethyl substituent. We applied this method to determine the stoichiometry of the adduct of pinacolyl methylphosphonate (PMP) and europium (III) tris naphthoyltrifluoroacetone (Eu(NTFA)_3_) in chloroform [17]. The excitation wavelength was 360 nm and the slits of the excitation and emission monochromators were set at 2 nm. A triangular cell, instead of the 180° geometry used by Brittain, was used to reproduce the experiment in a commercial fluorimeter. So, instead of 3.0 mL aliquots, 1.5 mL aliquots were employed. The spectra produced by the addition of partial equivalents of PMP to Eu(NTFA)_3_ up to 1.05 equivalents is presented in Figure 7 [17]. Unlike the phosphate esters studied by Brittain, addition of PMP beyond one equivalent causes a loss of β-diketone, as seen in Figure 8. Thus, instead of reaching a sustained maximum intensity of luminescence, the luminescence increases up to one equivalent and then decreases. This suggests that at high concentrations, the phosphonate could displace a β-diketone ligand. This behavior was not observed with the polymers, likely due to crosslinked site’s pseudo higher order chelation. The plot does show that the 1:1 complex has the greatest luminescence intensity.

**Figure 9:**
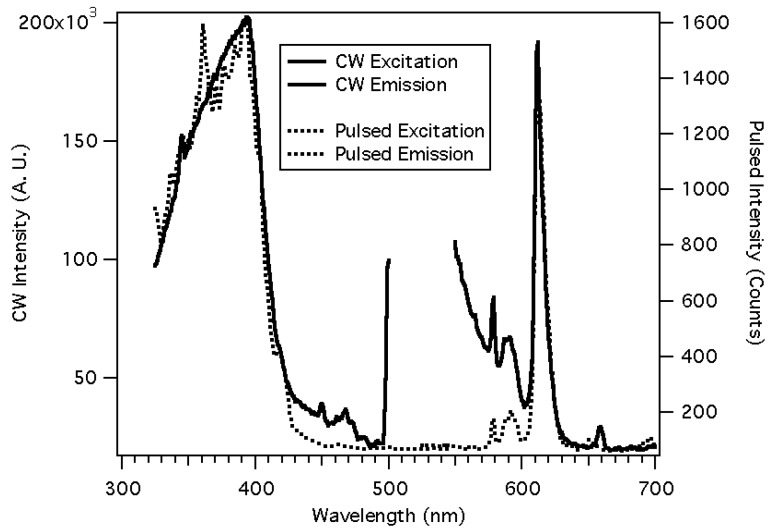
The use of time resolved luminescence to discriminate against background luminescence and scattered light [17].

The inclusion of a luminescent chromophore into an organic polymer may be complicated by background luminescence. When Eu(NTFA)_3_ was incorporated into a styrene copolymer, the continuous wave (CW) luminescence spectrum showed a large background, Figure 9 [17]. This background is observed when using a broad-band light source for excitation and not observed when the luminescence is excited by an Ar ion laser at 465.8 nm. Since our ultimate intent is to make small portable sensors, the ability to pump a broad allowed ligand band with a small light source such as a light emitting diode (LED), as opposed to pumping a sharp weak europium (III) absorbance with a large laser, is desired. The background was eliminated by using a pulsed light source and gated detection. The excitation spectra are not very different except there are some spikes due to the pulsed lamp’s output. The emission spectrum now has a flat background, showing the absence of scattered light and matrix fluorescence. The time-resolved spectra were obtained by delaying signal collection until the excitation light had been off for 30 microseconds and integrating for a period of 1 millisecond. The slits of the excitation and emission monochromators were set at 2 nm, and the polymers were mounted on glass plates, cut to fit the cuvette holder of the phosphorimeter. The long luminescence lifetimes exhibited by the lanthanide are compatible with less expensive and relatively slow electronics.

### 1.5. RAFT Polymers

**Figure 10:**
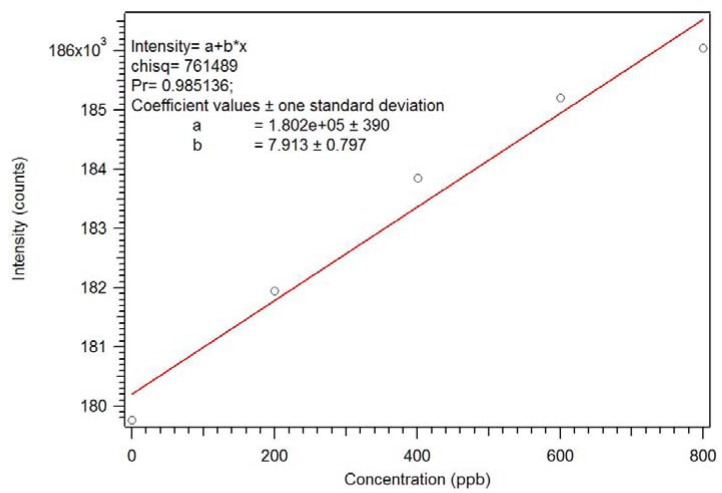
Calibration curve for the RAFT PMP MIP [17].

Initial cleaning was attempted using three refluxing solvents: acetone, methanol and isopropanol [17]. Isopropanol had the highest concentration of PMP after cleaning and was used in all subsequent cases. In a first study, five milligrams of the isopropanol-cleaned polymer was placed in a cuvette in xylene and examined by luminescence spectroscopy. Xylene was used as solvent as it has the same refractive index as polymer and roughly the same density. Ten-minute intervals were used between additions. A calibration curve generated by this process is presented as Figure 10. The curvature of the calibration curve suggested that either there were insufficient sites in the polymer to cover the dynamic range or that ten-minute intervals were not long enough to reach equilibrium.

**Figure 11:**
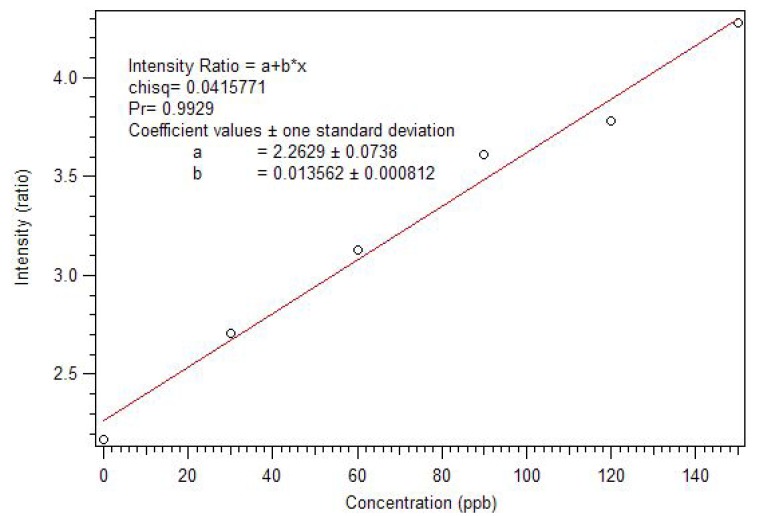
Calibration curve using longer time intervals and a ratio to standard showing much improved sensitivity [17].

In order to assure complete removal of PMP, the polymer was next cleaned by Soxhlet extraction with isopropanol. The concentration of PMP in the solvent was measured hourly until a steady state concentration was achieved. Extraction of the RAFT polymers does not liberate any europium, showing that the complexes are fully incorporated into the polymer. Since the incorporation of complex is better, smaller amounts of polymer can be used to get good sensitivity and dynamic range. A second calibration curve was generated using one and a half milligrams of the RAFT polymer prepared with methoxyethanol as solvent porogen. Also, a control solution of the model compound was used to adjust for instrument changes Figure 11.

### 1.6. Selectivity

The selectivity of the polymers was tested by luminescence titration using chemically similar organophosphates. The most likely interferent would be an organophosphate that is smaller than the imprint molecule so that it could fit in the imprinted site. With this in mind, two small organophosphates, dimethyl methyl phosphonate and dimethyl hydrogen phosphite were tested as interferences. Since these compounds usually have some moisture, after solutions were prepared in xylene, a small amount of anhydrous calcium carbonate was added to the stock. There was no appreciable change in signal, even when large concentrations of interferent were added [17].

**Figure 12:**
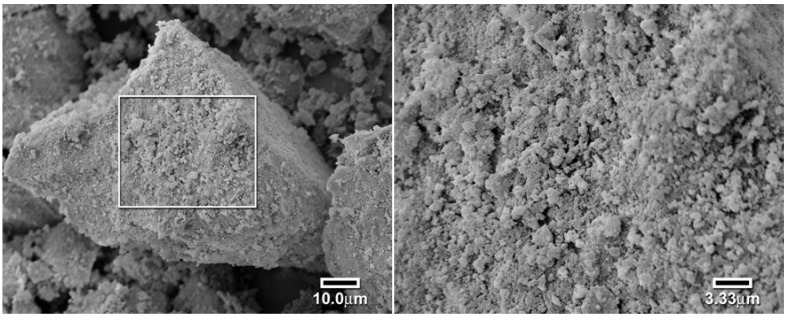
Scanning electron micrographs of a large particle of the mixed ligand polymer (left 1000×, right 3000×) [17].

**Figure 13:**
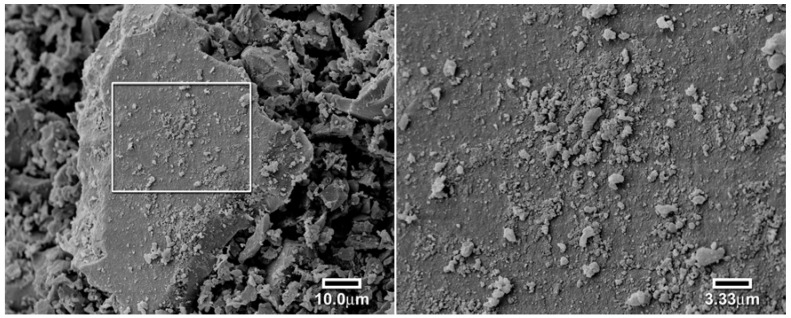
Scanning electron micrograph of a large particle of the RAFT polymer using toluene as the solvent (left 1000×, right 3000×) [17].

**Figure 14:**
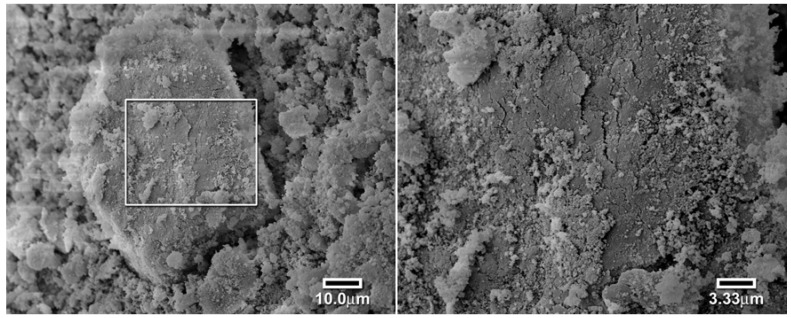
Scanning electron micrograph of a large particle of the RAFT polymer using methoxyethanol as the solvent (left 1000×, right 3000×) [17].

The slow kinetics associated with the first RAFT polymers was suspected to be caused by a lack of porosity or surface roughness. This hypothesis was tested by the use of a scanning electron microscope to investigate the particle’s surfaces. As seen in Figure 12, the conventional polymer particles have a rough surface conducive to fairly rapid equilibration [17]. However, the surface of the RAFT polymer is much smoother, as seen in Figure 13. This is attributed to the much more even and controlled reaction of RAFT polymerization. In order to improve the surface roughness and porosity, we changed the porogen/solvent to methoxyethanol, a highly polar solvent to induce phase separation during polymerization, Figure 14. The particles of the new solvent polymer were smaller. The RAFT polymer prepared using methoxyethanol was observed to form an opaque light pink polymer that readily cracked when solvent was removed. The polymer ground to a very fine powder and appeared to completely dissolve in xylene, showing that the refractive indices were matched.

Polymers prepared by grinding monoliths into particles were capable of sensing organophosphates at reasonably low levels and with good selectivity. Yet, the polymers were slow to respond and required a specific solvent with near equal density and the correct refractive index. It would be preferable to have particles small enough to be soluble and with little intervening polymer matrix to hamper diffusion. Since RAFT polymerization allows the formation of block copolymers, it is possible to prepare star polymers with crosslinkable cores and solubilizing “arms”.

### 1.7. Synthesis of Star Polymer Cores

**Figure 15:**
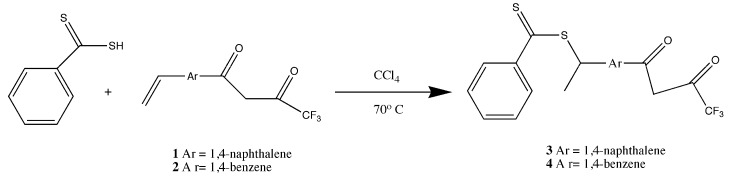
Preparation of the dithiobenzoate β-diketone ligands 3 and 4 [17,18,19].

The star polymer core consisted of a dithiobenzoate substituted tris(β-diketonate) europium (III) complex that was prepared in two steps. The dithiobenzoate β-diketone ligands **3** and **4** were prepared by the condensation of equimolar amounts of dithiobenzoic acid with a vinyl-substituted β-diketone in carbon tetrachloride at 70 °C (Figure 15). The corresponding tris Eu(III) chelates, **5** and **6** were prepared by the addition of the diketonate anion, to 0.33 equivalents of EuCl_3_(aq) and refluxing for 3 hours (Figure 13) [17,18,19]. 

### 1.8. Polymerization

Previous work with bulk polymers prepared from styrene, divinylbenzene, and **5**, with toluene as a porogen, and Wako V-65 as initiator are described above [17]. The solutions were polymerized at 60 °C for 18 hours. Cooling to room temperature stopped the polymerization followed by removal of toluene and the unreacted monomer *in vacuo*. The monolith was ground to a powder and washed with methanol and acetone to remove unreacted monomers and high boiling solvent. The method showed good incorporation of luminescent complex with no evidence of Eu(III) luminescence in the wash solutions. The gel effect was shown to be mitigated as demonstrated by their homogeneity. However, these polymers still required preparation *in situ* for sensor fabrication or application to a surface. Additionally, it was desirable to crosslink the volume immediately surrounding the lumiphore to enhance its stability in the melt or in solution.

In this work, soluble, unimolecular, luminescent polymers were prepared by RAFT polymerization [18]. The initial polymer used **5** as the core, which served as the polymerization substrate for the three-armed RAFT mediated polymer. The arms were AB block copolymers where block A was 1-but-3-enyl-4-vinylbenzene and block B was styrene, which imparts solubility upon subsequent intramolecular crosslinking by Ring Closing Metathesis (RCM) [39]. The monomer, 1-but-3-enyl-4-vinylbenzene, was chosen since it contains two reactive groups, which have different reactivities. The vinyl groups are polymerizable under free-radical conditions, while the allylic, but-3-enyl moiety remains stable under such conditions. However, the but-3-enyls are reactive under RCM conditions with second generation Grubb’s catalyst which give an intra-molecularly crosslinked core [40,41]. 

**Figure 16:**
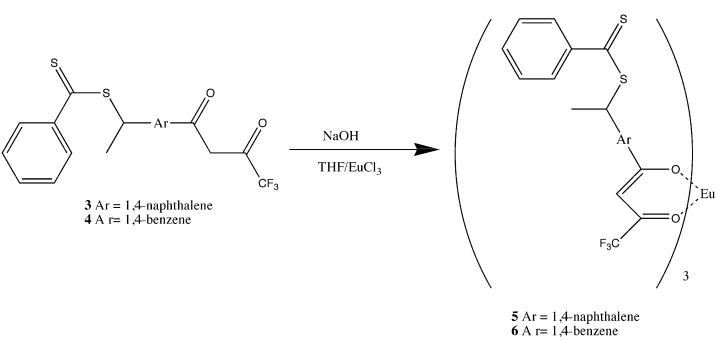
Preparation of the tris Europium core complexes **5** and **6** [17,18,19].

**Figure 17:**
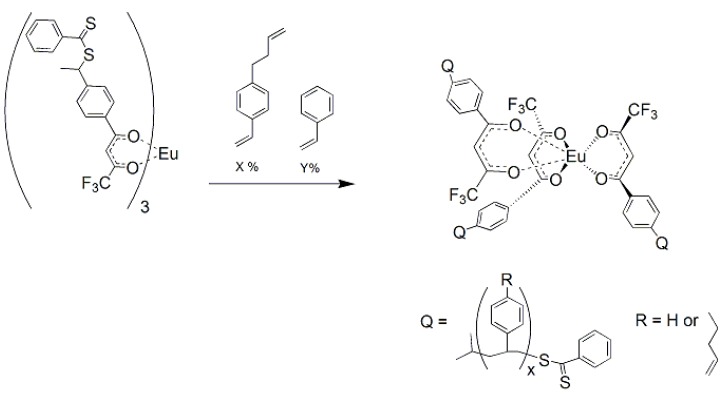
General synthetic scheme for Block A formation using core **6** [18].

P-1_A_ was prepared by addition of 1-but-3-enyl-4-vinylbenzene to **5**, with 2,2’-azobis(2,4-dimethylvaleronitrile (AIBN) as initiator, at 50 °C for 7 h (Figure 16) [18]. The polymerization was stopped by cooling the polymerization flask in an ice bath. Approximately 10% of the monomer was consumed, which would correspond to addition of seven monomers/arm of the CTA substrate. P-1_AB_ was polymerized at 60 °C for 72 h followed by 100 °C for another 24 h, and the polymerization stopped by addition to an ice bath after 70% of the monomer, styrene, was consumed (Figure 17). The M_n_ of P1-_AB_ was found to be 14,500 g/mol, M_w_ = 37,000 g/mol, and MWD = 2.5 by GPC analysis (Table 1). Luminescence titration of an aliquot of the uncrosslinked polymer with dimethylhydrogen phosphonate resulted in a molecular weight of 30,000 g/mole consistent with GPC analysis. Uncrosslinked polymers based on this formulation will interact with most phosphonates without selectivity.

**Table 1:** Properties of Soluble and Processable MIPs [18].

Polymer	%1-but-3-enyl-4-vinylbenzene	Block A, time ^a^	% conv A ^b^	Block B, time ^c^	MW	Mn	MWD
P-1_AB _^d^	100	7^f^	10	^g, h^	37,000	14,500	2.5
P-1_CR_					56,800	18,400	3.1
P-2_AB _^e^	3	7.5 h	40	8 h	33,900	26,600	1.28
P-2_CR_					35,900	26,000	1.38
P-3_AB _^e^	30	50 h	40	8 h	15,100	4,600	3.28
P-3_CR_					15,300	5,200	3.23
P-4_AB _^e^	100	50 h	41	8 h	23,200	6,500	3.58
P-4_CR_					23,300	6,900	3.38

^a^ Conditions: No initiator, 100 °C; ^b^ determined gravimetrically; ^c^ Conditions: AIBN, 60 °C; ^d^ Core is **5**; ^e^ core is **6**; ^f^ Conditions: Wako V-65, 50 °C; ^g^ Conditions: 60 °C 72 h, followed by 100 °C, 24 h; ^h^ Block B is Styrene.

**Figure 18:**
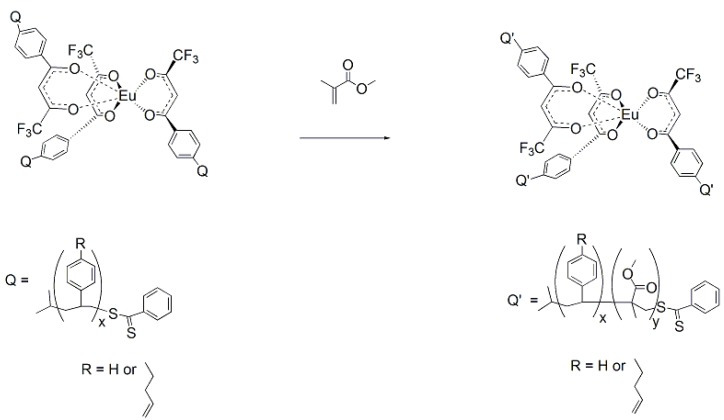
General synthetic scheme for Block B formation using methyl methacrylate for making polymer “arms” with core **6** [18].

P-1_AB_ was intramolecularly crosslinked in dilute methylene chloride under RCM conditions with second generation Grubbs catalyst, benzylidene[1,3-bis(2,4,6-trimethylphenyl)-2-imidazolidinylidene]dichloro-(tricyclohexylphosphine)ruthenium (Figure 18). The RCM crosslinked polymer, P-1_CR_ was determined to have a M_n_ = 18,400 g/mol, M_w_ = 56,800 g/mol, and MWD = 3.1 by GPC analysis. It is clear that some intermolecular crosslinking occurred during RCM, however intramolecular crosslinking predominated as evidenced by the relatively small change in molecular weight. 

**Figure 19:**
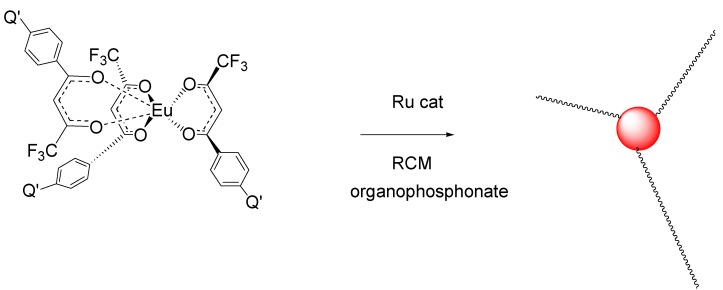
General scheme for crosslinking of the polymeric core using a second generation Grubbs catalyst [18].

**Figure 20:**
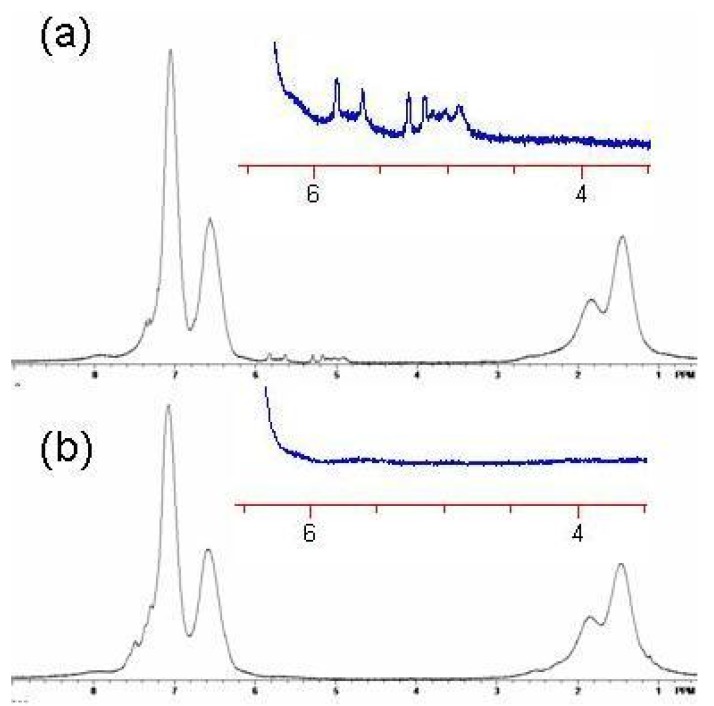
^1^H NMR spectrum of Polymer 1. (**a**) Before crosslinking by ring closing metathesis (RCM). Note the presence of the vinyl absorbances of the butenyl moiety between 4.8 and 6 ppm; (**b**) After crosslinking by RCM. Note the absence of the vinyl absorbances of the butenyl moiety between 4.8 and 6 ppm [18].

Crosslinking is consistent with the loss of free butenyl groups in the ^1^H-NMR. The free butenyls of the uncrosslinked polymer were found to have peaks from 5–6 ppm, but the peaks disappeared upon reaction with RCM catalyst (Figure 19 and Figure 20). Unexpectedly, the RCM catalyst also cleaved the dithiothioester end groups from the macromolecule as seen through the loss of the polymers salmon color. Others have also reported the loss of color due to dithioester cleavage [40,41]. The crosslinked polymers were soluble in methylene chloride, and chloroform.

**Figure 21:**
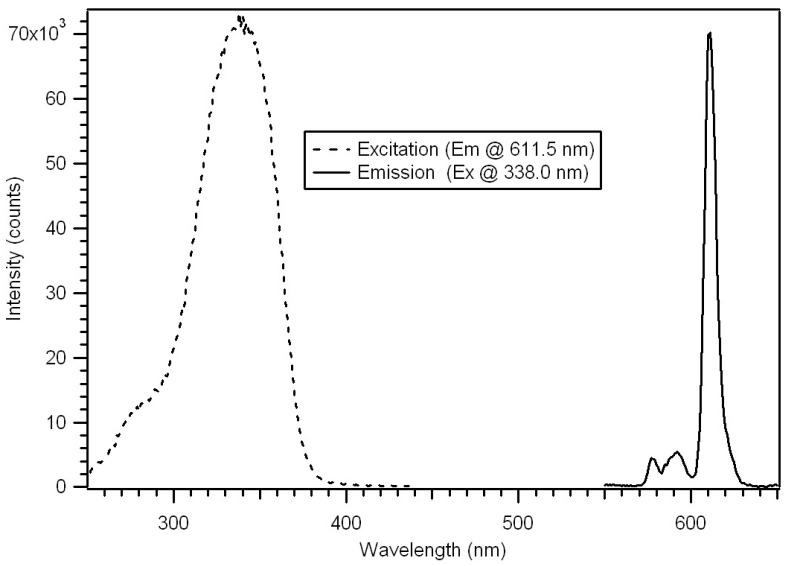
Pulsed excitation and emission spectra of P-4_AB_ with absorbance maximum 338 nm and emission maximum at 611 nm [18].

The progress of the polymerization was also studied by fluorescence [18]. Complexes **5** and **6** were weakly fluorescence due to the very strong dithioester chromophore absorbing the excitation wavelength of 390 nm. The polymerization reaction is visually evident by illumination with a long wave UV Lamp, revealing an increase in luminescence intensity during the reaction. The luminescence of the polymer increases dramatically as the distance between the dithioester chromophore and the europium increases. The luminescence of the star polymers is extremely bright. The pulsed gated spectra of 95 μg/mL of Polymer P-4_AB_ in CH_2_Cl_2_ are given in Figure 21. The spectra were obtained using a 20 μsec delay and 100 μsec integration times.

The polymers 1-4_AB_ were crosslinked with bound dicrotophos through RCM. It was impossible to remove the dicrotophos in polymers **1** and **4**, even with extensive Soxhlet extraction, since the core was 100% 1-but-3-enyl-4-vinylbenzene and completely crosslinked. In order to give some degree of freedom to the crosslinked core, polymers in which Block A was a copolymer of 1-but-3-enyl-4-vinylbenzene and styrene were prepared. A new core complex, **6** was prepared to ease of synthesis of **4** and stability of its precursors. Varying the degree of crosslinker in these polymers led to the preparation of luminescent, soluble and processable imprinted polymers (Table 1). All of the polymers have a narrow molecular weight distribution. Polymer **1** was the first star MIP prepared. The MW of the crosslinked polymer increased significantly along with the polydispersity. This was due to running the crosslinking reaction in a solution that was too concentrated, resulting in intermolecular crosslinking. In polymers **2**–**4**, the polydispersities are similar between the uncrosslinked and crosslinked polymers. As stated previously, uncrosslinked polymers exhibited no discernible selectivity and responded roughly the same to a variety of phosphonates.

### 1.9. Binding and Interference Studies

**Figure 22:**
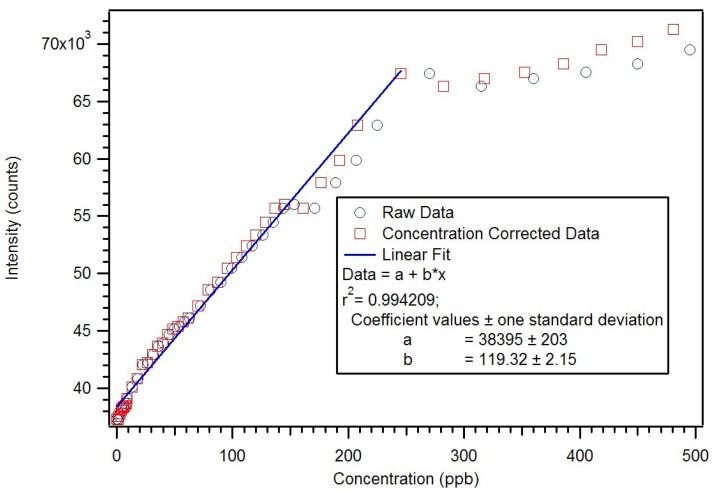
Luminescence titration of 0.1 mg/mL star MIP P-3_CR_ with 0.10 mM dicrotophos [18].

**Figure 23:**
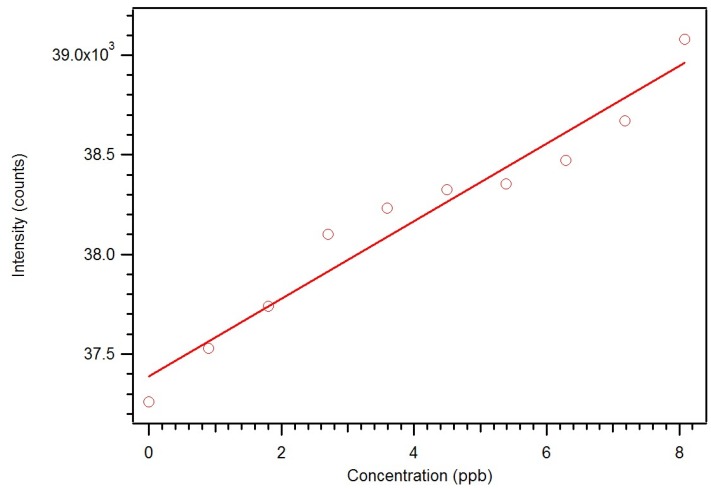
Expansion of the luminescence titration of 0.1 mg/mL star MIP P-3_CR_ with 0.1 mM dicrotophos showing a sub ppb detection limit [18].

Since the core of the star MIP has a lanthanide ion chromophore, luminescence can be used to verify that the polymer has useful properties. We have demonstrated that many organophosphates bind to tris(β-diketonate)Eu(III) complexes with a 1:1 stoichiometry [19]. The rebinding of dicrotophos to the P-4_CR_ was evaluated [18]. The Star MIP was dissolved in methylene chloride and aliquots of a dicrotophos solution were added. As seen in Figure 22 and Figure 23, this polymer can be used as a sensor with a sub ppb detection limit. As expected, an analogous non-imprinted polymer (NIP) showed little response with the addition of the dicrotophos solution, likely due to steric hindrance since they responded better to lower molecular weight phosphonates.

**Figure 24:**
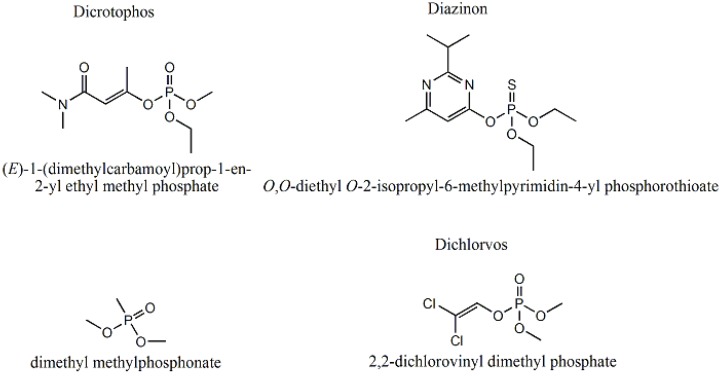
Structure of dicrotophos and tested potential interferents [18].

**Figure 25:**
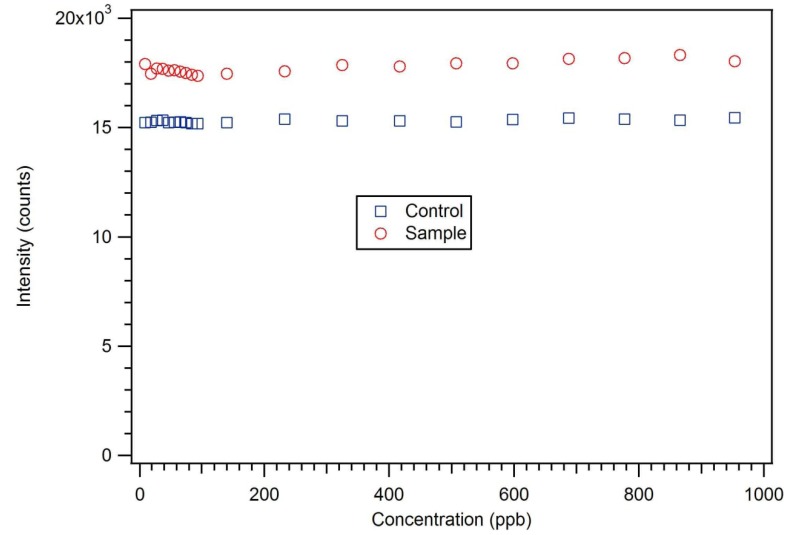
A dichlorvos luminescence interference test with star MIP P-3_CR_ shows essentially no response up to 1 ppm or 1,000 times the detection limit for dicrotophos [18].

P-3_CR_ was also highly selective for dicrotophos. Interference studies were performed using dichlorvos, diazinon, and dimethyl methylphosphonate (Figure 24). The insecticide that is most similar to dicrotophos is dichlorvos. Diazinon was selected due to the presence of nitrogen in the structure that might influence metal ion coordination. Dimethyl methylphosphonate was selected since it is a common organophosphate and its small size suggests it might be able to occupy a site imprinted with a larger molecule having similar structure. In these tests, no interference was detected from any of the three compounds, even when the polymer was subjected to concentrations 100 to 1000 times higher than the concentration of dicrotophos (Figure 25). The data for diazinon and dimethyl methylphosphonate were similar.

## 2. Conclusions

Molecularly imprinted polymers with good sensitivity, limits of detection (LOD) in the ppb range and very high selectivity (no interference detected from chemically similar interferents) have been prepared using bulk RAFT polymerization. The polymers were designed to be used in a liquid light guide based sensor system. The methacrylate polymers have the same refractive index as xylene and both the polymer and xylene should work well in a Teflon AF light guide. Polymers prepared using methoxyethanol have better porosity and exchange kinetics than polymers prepared using toluene as solvent and porogen. 

Soluble and processable molecularly imprinted polymers with good sensitivity (LOD’s in the low ppb range) and very high selectivity (no interference from near identical interferents) have been prepared using RAFT polymerization followed by Ring Closing Metathesis (RCM). The polymerization was done in the presence of a template to generate a processable star MIP. The core of the star polymer was a dithiobenzoate substituted tris(β-diketonate) europium(III) complex. The tris(β-diketonate) europium complex served as a polymerization substrate for the three armed RAFT mediated star polymer and as a luminescent binding site for dicrotophos, an organophosphonate pesticide. The star arms were AB block copolymers. Block A was either 1-but-3-enyl-4-vinylbenzene or a mixture of 1-but-3-enyl-4-vinylbenzene and styrene. Block B was styrene or methyl methacrylate. The but-3-enyls of block A were reacted by RCM with a second generation Grubbs catalyst to give an intramolecularly crosslinked core. The polydispersities of the polymers were initially high (for example, P-1_CR_, MWD = 3.1) due to interstar crosslinking. By crosslinking in very dilute solution the polydispersity improved (P-2_CR_, MWD = 1.38). The intramolecularly crosslinked MIP was soluble in common organic solvents. The 30% crosslinked soluble and processable star MIP was applied to the determination of dicrotophos with sub ppb level detection limits. The process results in macromolecules with terminal thiol groups amenable to binding to gold. The soluble MIPs are a powerful step forward toward the production of synthetic antibodies, improved chemical sensors, or highly stable and efficient luminescent plastics.
